# A 17-year-old boy with hemifacial flushing and anhidrosis

**DOI:** 10.1016/j.jdcr.2026.02.002

**Published:** 2026-02-06

**Authors:** Pedro Simões Farinha, Ana Ferreirinha, Beatriz Vilela, Bruno Duarte

**Affiliations:** a Department of Dermatology and Venereology (CRI de Dermatovenereologia), Hospital de Santo António dos Capuchos, ULS São José, Lisbon, Portugal; b NOVA Medical School (NMS), Faculdade de Ciências Médicas (FCM), Universidade NOVA de Lisboa, Lisbon, Portugal

**Keywords:** case report, glycopyrronium, Harlequin syndrome, hyperhidrosis, oxybutynin

## Case description

A 17-year-old boy presented with a long-standing history of episodic unilateral flushing and hyperhidrosis involving the left hemiface, scalp, and shoulder, first noted at age 2. Episodes were consistently triggered by heat, exercise, or emotional stress and were accompanied by contralateral (right-sided) anhidrosis/hypohidrosis, creating a striking midline asymmetry. He denied any neurologic symptoms, trauma, or previous surgeries. Neurologic examination was normal, and magnetic resonance imaging of the brain and cervicothoracic spine excluded structural or compressive lesions.

**Fig 1:**
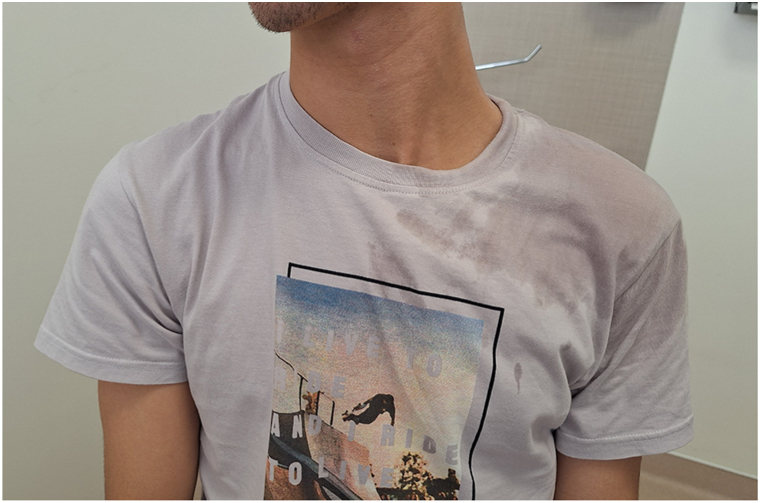
Initial presentation showing a soaked T-shirt from severe hyperhidrosis with evident unilateral flushing and sweating involving the left cervical and shoulder regions.

**Fig 2:**
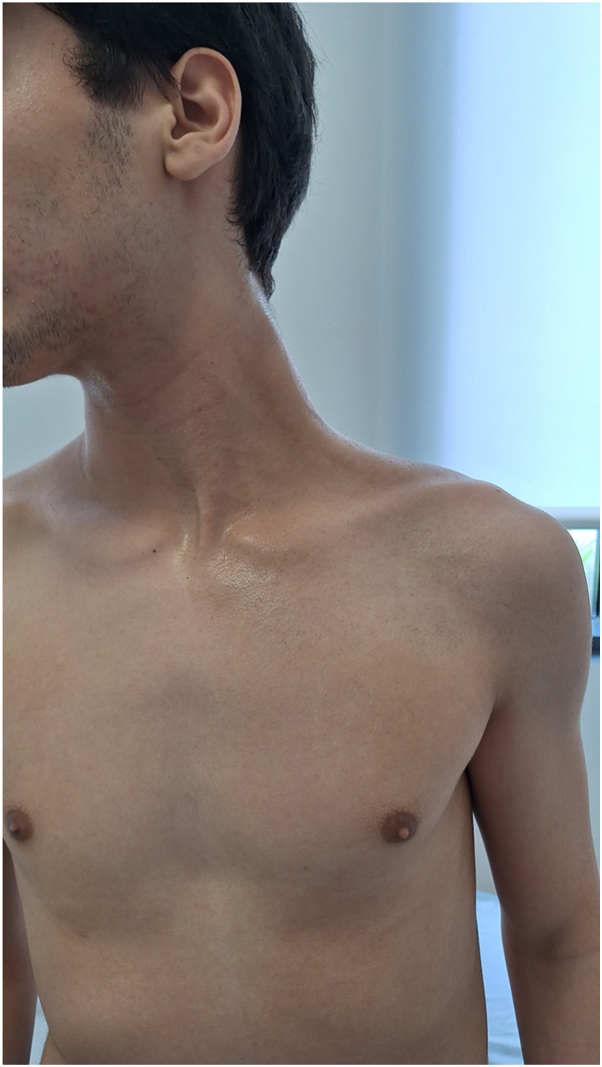
Close-up view highlighting asymmetric vasodilation and hyperhidrosis of the left cervicoscapular area.

At presentation, the patient exhibited marked hyperhidrosis with visible soaking of clothing and pronounced flushing of the left cervical and shoulder regions (Fig 1). A close-up view demonstrated distinct asymmetry in vasodilation and sweating, with relative dryness on the right side (Fig 2).

Due to significant psychosocial distress and interference with daily activities, pharmacologic treatment was initiated. Oral oxybutynin was started at 2.5 mg nightly and titrated to 5 mg at midday and 2.5 mg at night. Topical glycopyrronium 8 mg/g cream was applied daily for 1 month, then reduced to 3 times weekly. After 2 months, complete remission of symptoms was achieved, and the patient remained asymptomatic at 12-month follow-up without adverse effects.

**Question: Which of the following is the most likely diagnosis?**

**A.** Segmental facial rosacea
**B.** Frey syndrome
**C.** Harlequin syndrome (HS)
**D.** Tonic pupil with facial flushing
**E.** Chorda tympani syndrome

**Answers**

**Correct answer: (C) HS.**

HS is a rare autonomic disorder characterized by episodic, sharply demarcated hemifacial and cervicothoracic flushing and sweating, typically precipitated by exertion, heat, or emotional stress. HS results from unilateral disruption of sympathetic vasomotor and sudomotor pathways. The affected side demonstrates reduced or absent flushing and sweating (anhidrosis/hypohidrosis), whereas the contralateral, unaffected side exhibits flushing, hyperemia, and sweating that may appear exaggerated (“compensatory”) during triggers. Although most cases are idiopathic, secondary causes—such as trauma, space-occupying lesions, neurodegenerative diseases, or surgical injury—must be excluded through neuroimaging.^1^

In this patient, the long-standing, heat- and stress-induced left-sided flushing and hyperhidrosis with contralateral (right-sided) anhidrosis, absence of neurologic deficits, and normal brain and cervicothoracic magnetic resonance imaging supported a diagnosis of idiopathic HS. While HS is benign, it can cause profound psychosocial distress, particularly in adolescents.^2^

Anticholinergic therapy is a rational approach, as it suppresses sweat gland activity through muscarinic receptor blockade. Oxybutynin acts systemically on M3 receptors, whereas topical glycopyrronium inhibits M1 and M3 receptors locally. This combination optimizes sudomotor inhibition with minimal systemic side effects. To our knowledge, this is the first reported case of idiopathic HS successfully treated with dual systemic and topical anticholinergic therapy, achieving durable remission without complications.^3^

Alternative treatments include botulinum toxin injections for localized hyperhidrosis,^4^ though large treatment areas and repeated sessions are often required. Invasive interventions such as stellate ganglion block or thoracic sympathectomy^5^ have variable efficacy and carry procedural risks. This case demonstrates a safe, accessible pharmacologic approach for symptom control in HS and underscores the importance of recognizing this distinctive yet underdiagnosed condition.

## Conflicts of interest

None disclosed.
